# The Insanity of King George III
*Read before the Association of Superintendents of Insane Hospitals, May 22, 1855. By Dr. Ray, of Butler Hospital, Providence, R. I.—*From the American Journal of Insanity*.


**Published:** 1857-01-01

**Authors:** 


### Art. VII.—THE INSANITY OF KING GEORGE III.*

To the mere pathologist, the insanity of a prince is not more interesting than that of a peasant; but to the historian, to the medical jurist, to all who are engaged in the care of the insane, the attacks of George III. are invested with peculiar interest. He was a prominent figure in a period that teemed with great men and great events, whose memorials are yet around us; and twice the recurrence of his disorder gave rise to a degree of political feeling that has seldom been equalled, and to political discussions that settled for ever a vital principle in the British constitution.

George III. had a moderate intellectual capacity, but an obstinate will. Of abstract speculation he was totally incapable, and philosophical views of any kind were beyond his reach. His theory of government began and ended in a firm maintenance of the royal prerogative, and the whole duty and privilege of the subject were comprised in the single precept, *Fear God and honor the King*. As a result, partly of defective training and partly of original inaptitude, he disrelished intellectual

* Read before the Association of Superintendents of Insane Hospitals, May 22, 1855. By Dr. Ray, of Butler Hospital, Providence, R. I.—*From the American Journal of Insanity.*

pursuits, but was fond of mixing himself up with the administration of affairs, even in the smallest particulars. Here he showed no lack of industry, nor of energy. He was a stranger to sensual passion, and in the common observances of life was a model of propriety. He never forgot what he deemed an injury, and they who thwarted his wishes or opposed his measures were regarded as factious or dishonest. Always looking upon his eldest son as a kind of rival near the throne, "he hated him," says Brougham, "with a hatred scarcely consistent with the supposition of a sound mind." He was fond of music, and occasionally went to the theatre; but, with these exceptions, he sought for recreation solely in riding and walking, in looking after his farm, and in an easy intercourse with his family and dependents. Few men would have seemed less likely to be visited by insanity. His general health had been always good; his powers were impaired by none of those indulgences almost inseparable from the kingly station; he was remarkably abstemious at the table; and took much exercise in the open air. Insanity had never appeared in his family, and he was quite free from those eccentricities and peculiarities which indicate an ill-balanced mind.

Five times was George III. struck down by mental disease. The first was in the spring of 1765, when he was twenty-seven years old; the second in 1788; the third in 1801; the fourth in 1804; and the fifth in 1810. Excepting the last, from which he never recovered, the attacks were of comparatively short duration, none of them continuing very obviously beyond six months.

The particulars of the first attack were studiously concealed by his family, and its true character was not generally known at the time. There seems to be no doubt, however, that its symptoms were similar to those of the subsequent attacks. Shortly before, an eruption on the face, which had troubled him for some years, had so entirely disappeared, that it was supposed he had applied external remedies to repel it. This was followed by considerable cough and fever, and then by mental disturbance. In the course of a few weeks he completely recovered.

During the latter part of October, 1788, the King seemed to be not in his usual health. He had considerable pain in his limbs—felt weak—slept but little—was hurried and vehement in his manner. On the 22nd, he "manifested an agitation of spirits bordering on delirium," said his physician. A few days afterwards, on returning from a long ride, he burst into tears, and said, "he wished to God he might die, for he was going to be mad." He kept about until the 4th of November, when he had an outbreak at dinner, and was consigned to the charge of attendants. During the first few days there was considerable constitutional disturbance, and it was feared he might not survive. One of Sheridan's correspondents says: "The doctors say it is impossible to survive it long, if his situation does not take some extraordinary change in a few hours. * * * Since this letter was begun, all articulation even seems to be at an end with the poor King; but, for the two hours preceding, he was in a most determined frenzy." In the course of the succeeding night he had a profuse stool, then perspired freely, and fell into a profound sleep. He awoke with but little fever, "but with all the gestures and ravings of the most confirmed manaic, and a new noise in imitation of the howling of a dog." He soon got calmer, and talked on religion, and of being inspired. A day or two after, the same person writes: "This morning he made an attempt to jump out of the window, and is now very turbulent and incoherent." He also states that the King revealed some state secrets, much to the astonishment of Pitt.*

Miss Burney, afterwards Madame D'Arblay, was then in the personal service of the Queen, and in her "Diary," recently published, the progress of the attack may be traced with some degree of minuteness. The first night after the outbreak at dinner, she states, he was very restless, getting up and wandering into the Queen's room to see if she was there, and talking incessantly until he became hoarse, exclaiming, "I am not ill; I am only nervous." "He was never so despotic; no one dared oppose him. He would not listen to a word." Next night he got up and insisted on going into the neighbouring room, where his equerries were. There he saw his physician, Sir George Baker, whom he called an old woman, and wondered that he ever took his advice, for he knew nothing of his complaint. From this time he rapidly grew worse. On the 12th and 13th of November he appeared considerably better, and continued so until the 20th, when he became as bad as ever. From this period his condition was variable—always more or less excited—rather petulant, if not irascible—scolding his gentlemen for slighting him. On the 29th of November he was removed to Kew, where were better opportunities for exercise. Through the month of December there was little, if any, change in his condition. During the first two or three weeks in January he became less irritable, was quite calm at times, and then would read and make sensible remarks on what he had read. From the latter part of the month he steadily improved. February 2nd, Miss Burney accidentally saw him walking in the garden, and, to avoid meeting, in compliance with the rules, ran off at full speed, and he after her, the physicians and attendants in

* Moore's Life of Sheridan, p. 360. Amer. edition.

full chase after him. She finally stopped until he came up, when he put his arms around her neck and kissed her. He talked incessantly, blurting out whatever came uppermost. "He seemed to have just such remains of flightiness as heated his imagination without deranging his reason, and robbed him of all control of his speech, though nearly in his perfect state of mind as to his opinions. * * He opened his whole heart to me, expounded all his sentiments, and acquainted me with all his intentions." He declared he was as well as he ever was in his life—talked of the official situation of her father, of music, (when he undertook to sing,) and then of her friends. He said he was dissatisfied with his ministers, and showed a list of new ones he had prepared. On the 17th he received the Chancellor, on the 18th drank tea with the Queen, and on the 7th of March received the Address of the Lords and Commons in person.*

One of his first excursions was to a poor-house in the course of erection, of which he inspected every part, especially the rooms for lunatics, and expressed much satisfaction that such excellent accommodations were provided for persons labouring under the misfortune of insanity. During his convalescence, it is said, he passed much of his time in reading the debates on the Regency Bill.†

The King was attended, at first, by his own physicians, Sir George Baker and Dr. Warren, and they were, shortly after, joined by Sir Lucas Pepys, Drs. Reynolds, Gisborne, and Addington, of whom the latter alone had given any special attention to the treatment of insanity, and he discontinued his attendance after a few days. They had all achieved professional distinction, but Warren enjoyed an undisputed pre-eminence. He was not only at the head of his profession in London, and deservedly so, but such were his talents and manners that he associated intimately with the leading men of the day,—Burke, Fox, Sheridan, &c.—and was appointed physician to the Prince of Wales. The attack not readily yielding, it was thought proper by the Queen and the ministers, who had the direction of these matters, to have the constant attendance of some one particularly skilled in diseases of the mind. Their choice fell on the Rev. Dr. Francis Willis. This gentleman was educated for the established church, and took charge of a parish in Lincolnshire. Having some knowledge of medicine, he was fond of prescribing for the medical as well as the spiritual wants of his people, and especially for mental diseases. He was very soon regarded as very successful in this department of the healing art, and was so much resorted to, that he provided an

* Diary and Letters, ii. Phil., 1842.

† Wraxall's Posthumous Memoirs of his Own Time, p. 520. Phil.

establishment designed especially for the treatment of the insane. He was much patronized by the higher classes, and for fifty-eight years he had never less than thirty patients under his care. He was at this time seventy years old, but "seemed to be exempt from all the infirmities of old age, and his countenance, which was very interesting, blended intelligence with an expression of placid self-possession."* Miss Burney describes him as "a man of ten thousand, open, honest, dauntless, light-hearted, innocent and high-minded." He joined the corps of physicians on the 6th of December, and took up his quarters in the palace.† In the consultation which settled their respective functions, Willis was to have charge of all the domestic and strictly moral management—in accordance, however, with such general views as had been agreed upon. The medical treatment was arranged in the morning consultation, and it was understood that Willis was to take no decided measure, either medical or moral, not previously discussed and permitted. Pepys, Gisborne and Reynolds attended, in rotation, from four o'clock in the afternoon until eleven the next morning. Warren or Baker visited in the morning, saw the King, consulted with Willis and the physician who had remained over night, and agreed with them upon the bulletin for the day. Willis was soon joined by his son John, whose particular function seems not to have been very definitely settled. Willis professed to regard him as equal to himself in point of dignity and responsibility, but his colleagues considered him as merely an assistant to his father. Two surgeons and two apothecaries were also retained, each one, in turn, staying twenty-four hours in the palace. The personal service was rendered by three attendants, whom Willis had procured from his own establishment, and the King's pages,—one attendant and one page being constantly in his room.‡

* Wraxall, ibid. p. 447.

† Among the gossip of the court it was related that the "King asked Willis, when he entered the room, if he, who was a clergyman, was not ashamed of himself for exercising such a profession. 'Sir,' said Willis, 'our Saviour himself went about healing the sick.' 'Yes,' answered the King, 'but he had not 700*l.* a-year for it.'"—*Lord Malmesbury's Diaries, &c.*, iv. 317.

‡ The kind of supervision and attendance that was practised during this illness, and which was the same, probably, in the subsequent attacks, would seem sufficient to have prevented the slightest abuse of trust; and yet the King told Lord Eldon that, in one of his attacks, but which it does not appear, he was knocked down by a man in the employ of some of his physicians. "When I got up again," he added, "I said my foot had slipped and ascribed my fall to that; for it would not do for me to admit that the King had been knocked down by any one." [Twiss, i.—"Public and Private Life of Lord Chancellor Eldon.] We learn nothing further respecting this fact, and are left in doubt whether it actually occurred, or originated in that intellectual or moral obliquity, (almost universal among the insane, but the exact nature of which has never been thoroughly understood,) which leads them to exaggerate, distort, and pervert much that falls under their observation, and to fabricate much that never occurred at all. This curious trait of

The medical treatment seems to have consisted chiefly of "bark and saline medicines." An alterative pill, containing a little calomel, was given him once. Once, and once only, blisters were applied—to the legs—but they occasioned considerable irritation and restlessness.

It was determined that the moral management of the King required strict seclusion from his family and ministers, and, as far as possible, from all other company. But nothing can more strikingly indicate the change that has occurred since that time, in respect to one means of managing the insane, than the fact that, for two or three months, the King was frequently subjected to mechanical restraint. There was nothing, however, in his condition which would be considered at the present time a sufficient reason for its application. He was not disposed to injure his person or his clothing, his attendants or his furniture. In the King's case—and this, no doubt, was an example of the ordinary practice—it was evidently used by way of discipline, as a means of subduing turbulence and increasing self-control. Willis said, in his second examination by the Committee of the House of Commons, that when he took charge of the King, he was dissatisfied with the restraint which had been previously used, and for five days "endeavoured to persuade and explain," that some more efficient method would be resorted to, unless there was a "ready compliance" with his wishes. The King seems to have been insensible to this kind of intimidation, and the new mode of restraint was applied, with the effect, as Willis states, of accomplishing the desired purpose more effectively than before, being "more firm but not so teasing to the patient." It does not appear what means of restraint was used by Willis, or by the other physicians, but an incident related by Wraxall renders it probable that one of them was that time-honoured implement which is still associated with the popular idea of insanity. While walking through the palace, during his convalescence, accompanied by an equerry, they observed a strait-jacket lying in a chair. The equerry averting his look, as if to conceal some embarrassment, the King said: "You need not be afraid to look at it. Perhaps it is the best friend I ever had in my life."* This incident does not strengthen a favourite position of the advocates of non-restraint, that it leaves disagreeable impressions upon the patient's mind.

Of another fact respecting the King's treatment I cannot find a sufficient explanation. Between the 6th of December and the mental pathology deserves to be closely studied, not only because it is curious, but because it will be found, I think, to have some important bearing on human veracity, and human testimony in the normal state.

* Posthumous Memoirs, &c., p. 520.

13th of January he went out of doors but twice, and for a month previous not at all. Considering the form of the disorder and the facilities for exercise which the grounds afforded, this is certainly surprising. On one occasion, when the King had been promised a walk, Dr. Warren revoked the promise, because, as the day was cold, and the King had perspired freely in the night, there would be some risk of his taking cold.*

The political consequences of the King's illness proved to be of the deepest interest, whether we regard the magnitude of the questions at issue, or the men by whom they were discussed. The array of talent which distinguished the parliament of that period has never been equalled before or since. The interests of the administration were supported by Pitt, Thurlow and Wilberforce, while the forces of the opposition were led by Fox, Burke, Sheridan, Windham, Grey, Loughborough, and North. During the two or three months that the struggle lasted, every weapon of argument, wit, ridicule and invective was used by the contending parties with a dexterity and vigour which such men only could display.

When the King's incapacity was announced, parliament immediately set about to provide a regency. All parties agreed that the Prince of Wales should be the Regent, but differed very widely as to the exact amount of authority and privilege he should receive. The Whigs contended that he should exercise all the functions of the Sovereign precisely as if there was a demise in the Crown. The ministers, on the other hand, were determined to hamper the Regent with limitations and restrictions which would have shorn the regal office of much of its dignity and power. The real question at issue, therefore, was, which of the two parties that divided the country should possess the administration, and hence the violent party-spirit which characterized all the political proceedings of the time. The first step was to ascertain officially the exact condition of the King, and, accordingly, each House appointed a committee to examine his physicians. These committees performed the duties assigned them on the 10th of December, and their reports were laid upon the table a few days afterward.

To each physician was put the following questions: "Is his Majesty incapable, by reason of the present state of his health, of coming to parliament, or of attending to public business? What hopes have you of his recovery? Is your answer to this question founded upon the particular symptoms of his Majesty's case, or your experience of the disorder in general? Can you form any judgment or probable conjecture of the time his Majesty's illness is to last? Can you assign any cause for his illness? Do

* It appears that on that night the restraint had not been removed at all.

you see any signs of convalescence?" The replies to these questions evince a knowledge of insanity quite creditable to men not expressly devoted to this branch of the science—one that would hardly be expected by us who witness so frequently the remarkable discrepancies of opinion that characterize the reports of medical commissions, albeit they may include men whose names are not entirely unknown to fame. The replies also evince a certain kind of discretion and reserve worthy of all imitation on the part of those who are called upon for professional opinions. Few medical witnesses succeed, as most of these gentlemen did, in hitting that happy medium between saying too much and saying too little. They all expressed strong hopes of the King's recovery, because the majority of patients actually do recover, and they saw nothing particularly unfavourable in his case. None of them saw any signs of convalescence, and, with one exception, none of them pretended to assign causes or limits to his disorder. Willis said he would recover within a few months, and thought the attack was produced by "weighty business, severe exercise, too great abstemiousness, and little rest." The other physicians were as well aware as Willis, no doubt, of these facts in the history and habits of the King, and possessed better opportunities than he had of knowing how far they had affected his mind, but refrained from assigning them as causes of the disorder. Willis's opinion, though confidently uttered, was merely a speculation, resting on no very substantial grounds. The King's business had not been weightier than usual, and though fond of exercise, there is no evidence that he carried it to a degree incompatible with its proper object, the promotion of health. His abstemiousness consisted merely in avoiding that excessive indulgence in the pleasures of the table which was common among the higher classes of that period, and was practised by him for the purpose of warding off disease. The want of sleep was probably one of the effects rather than a cause of his mental affection. Whether the committee were satisfied with Willis's theory does not appear; but most of them probably were, like the rest of the world, curious to learn the cause of the attack, but readily satisfied with elaborate phrases and dogmatic assertions.* Sheridan, however, saw in it a fair mark for his wit, and he was not the man to neglect an opportunity of that kind. Willis had stated, in proof of the correctness of his opinion, that the medicine which had been given to his Majesty ever since Sunday morning, in order to meet and counteract those causes, had had as much effect as he could wish, and "his Majesty had certainly been gradually better from the first six hours of his taking it." The orator said that, when he heard Dr. Willis assert that his physic could, in one day, "overcome the effects of seven-and-twenty years' hard exercise, seven-and-twenty years' study, and seven-and-twenty years' abstinence, it was impossible for him to keep the gravity fit for the subject. Such assertions put him in mind of those nostrums that cure this and that, and also disappointments in love and long sea-voyages."*†

The policy of the cabinet was to make it appear that the King's illness would be of short duration, and let it be implied, as an obvious consequence, that the measure of appointing a Regent should not be precipitated. On the other hand, the policy of the Whigs was to represent the disorder as incurable, or, at least, of very uncertain duration, and therefore that the sooner the Regency was established the better for the country. In this view they received but feeble support, certainly, from the examination of the physicians; but Warren, who was high in the councils of the Whig party, had privately encouraged the idea that the King would never recover. True, in his examination just referred to, and also in the examination on the 7th of January, he expressed as much confidence as the others in his ultimate recovery. The fact furnishes a striking illustration of the distorted influence of party-spirit, even upon the views of scientific men on scientific subjects. Willis, who always professed to be quite sure of the King's recovery, and was equally high in the estimation of the other party, inspired the administration with confidence in the policy they had adopted. Every occurrence at Kew was whispered about in political circles, before it was many hours old, coloured and exaggerated, of course, by the prevalent hopes and fears. The names of Warren and Willis became as familiar as household words, and even served as rallying points for the two great parties that divided the country. In less than a month from the first examination, both parties were equally ready for another, and equally confident of deriving political capital from the result. For this purpose the Commons appointed a select committee, which commenced its sittings on the 7th of January, and made their report, 400 folio pages long, on the 14th.‡ The same questions as

* The Par. Debates on the Regency are contained in the 27th vol. of Hansard.

† The fact that the medicine referred to—which was simply Peruvian bark—was determined upon in the consultation of the whole corps of the King's physicians, and that no other observed any improvement in his condition, gives additional pungency to the ridicule, while the whole incident throws much light on Willis's character.

‡ The Report of the first examination may be found in the Parliamentary Debates before were put to the physicians, and were followed by the same replies, except that Willis, when asked if he had observed any signs of convalescence, replied affirmatively. The greater part of the examination was directed to matters having only an incidental connexion with the King's condition,—the communications sent from Kew to the ministers and other leading characters, the domestic arrangements of the palace, the dissensions of the physicians, the merits and proceedings of the Willises,—to anything, indeed, calculated to strengthen one side or weaken the other. Upon the signs of recovery or convalescence the examination was particularly searching, because, more than anything else, they determined the political movements of the day. Willis, when asked if he saw any present signs of convalescence, replied: "About a fortnight ago, his Majesty would take up books and could not read a line of them; he will now read several pages together, and make, in my opinion, very good remarks upon the subject. I think, in the main, his Majesty does everything in a more rational way than he did, and some things extremely rational." (This trait had been observed for the last five or six days, the books having been selected by the King, and read aloud.) To the same purpose, he also stated that his patient was less frequently and less intensely excited, and less frequently required restraint. Beyond the simple acknowledgment that he was more quiet, the other physicians were not supposed to go in regard to the signs of convalescence. They denied that he had appeared rational, even for a moment; but none of them had happened to see the King reading, and they were not disposed to take any fact of Willis's observing as a ground for their opinions. His constant attendance gave him an advantage over his colleagues, for it enabled him to see for himself much that they would never know at all, or only at second-hand; and such observations, we are all very well aware, sometimes leave a stronger impression on the mind than the most definite and tangible facts communicated by others.

Willis's character, conduct, and practices were subjected to a very searching scrutiny, not more for the purpose of obtaining information than of torturing every incident into matter of censure against himself or his employers. It cannot be denied that he gave his adversaries abundant opportunities of this kind; for, with all his experience, and the frost of seventy years on his head, he had not a philosophical turn of mind, nor the power of concealing his deficiency by a prudent reserve. He had stated and Annual Registers of the time, but not so this, which long eluded my search, until found in a collection of pamphlets, entitled, "History of the Regency," published by Stockdale, and brought to my notice by the librarian of Brown University, Mr. Guild. From this Report chiefly I have obtained all that seemed worth preserving respecting the management of the King.

that nine out of ten of his patients recovered under his hands, but he was unable to tell how many he had received or how many he had cured. When further pressed, he said that the ground of his calculation was the fact that his first fifteen patients were cured, and that, subsequently, several instances occurred of ten going away together radically cured! The declaration of his colleagues respecting this alleged success—that it required other evidence than his bare assertion—was not calculated to restore the harmony which had been so thoroughly disturbed. He was obviously very restive under the unusual restrictions imposed upon him. To be associated on equal terms with some half-dozen other physicians, equal to himself in professional eminence, and more than his equals in general culture, he found a very different position from that of controlling an establishment where his simple word was law. He felt—very correctly, no doubt—that a great obstacle to the King's recovery consisted in his being obliged to see so many different persons, under circumstances calculated to excite strong emotion. He was actually disturbed, and sometimes even prevented from sleeping, by the visits of so many medical men—never less than half-a-dozen every day—and, accordingly, Willis, "thinking it his duty," as he says, "to do for his Majesty what he should do for any private gentleman," put up a written notice that no person should be admitted into his Majesty's rooms without permission of himself or son. For this order, which was more easily given than enforced, for none of his colleagues seem to have regarded it, he was severely handled by the committee, who endeavoured to make it appear like an attempt on his part, and that of the Lord Chancellor, whose sanction he pleaded, to conceal, in some degree, the King's real condition.

Another obstacle to the King's recovery, apprehended by Willis, seems rather fanciful than real. "When his Majesty," he says, "reflects upon an illness of this kind, it may depress his spirits and retard his cure more than a common person;" but, subsequently, he states, that "this apprehension is somewhat relieved by his knowledge of the King's sense of religion, which may lead him, with a proper resignation, to reflect on what it had pleased God to afflict him with."

The want of good faith was broadly charged upon Willis by his colleagues, and in the examination there came out one instance of it which has obtained a popular celebrity. Warren stated, that, on the day Willis arrived, it was agreed, in general consultation, "that quiet of body and mind were to be endeavoured to be obtained by every means possible; and that everything should be kept from his Majesty that was likely to excite any emotion; that though his Majesty had not shown any signs of an intention to injure himself, yet that it was absolutely necessary, considering the sudden impulses to which his distemper subjects people, to put everything out of the way that could do any mischief." The very next day, however, he put into the King's hand a razor and a penknife. "I asked him," says Warren, "how he could venture to do such a thing. He said he shuddered at what he had done." Willis said, in explanation, that the King "had not been shaved for a long while, perhaps a fortnight or three weeks; and the person that had been used to shave him could not complete the parts of his upper and under lips; and being confident, from the professions and humour of his Majesty at that moment, I suffered his Majesty to shave his lips himself; and then he desired he might have his whole face lathered, that he might just run over it with a razor; and he did so in a very calm manner. His nails also wanted cutting very much; and, upon his assurance, and upon my confidence in his looks, I suffered him to cut his own nails with a penknife, while I stood by him. It is necessary for a physician, especially in such cases, to be able to judge, at the moment, whether he can confide in the professions of his patient; and I was never disappointed in my opinion whether the professions of the patient were to be relied on or no." He denied that he said to Warren, he shuddered at what he had done, and also denied that, in regard to such matters, he ever agreed not to be governed solely by his own discretion. After professing such views, he found it a little inconvenient to answer the question, why he never afterwards repeated this indulgence. He replied, however, that it had a bad moral effect, his Majesty taking it ill that he was not allowed other privileges, such as going up stairs to see his family, and doing other imprudent things. "Do you think," asked the committee, "that the expectation of the liberties which the King might call for would be of more danger to him than the use of razors and penknives?" "To be sure," was the reply, "because refusal would irritate him much and increase his disorder." "Whether," continues the committee, "you refuse to the King all indulgences which may be safely given, lest he should demand those that ought to be refused?" "I do a great many," said Willis. Those, certainly, were very embarrassing questions.

This incident furnished Burke with the materials of a violent diatribe against the ministers, who, he said, had committed his Majesty to the care of a man in whose hands he was not safe for a moment.*

* There is a traditionary anecdote connected with this razor scene, strongly illustrative, if true, of Willis's character. Burke asked him, it is said, what he would have done, if the King had suddenly become violent while these instruments were in his hand. Having placed the candles between them, he replied, "There, sir,

It also came out that, within five days after he took charge of the King, Willis allowed him to have an interview with his daughters, and another with the Queen, without the consent or knowledge of his colleagues, and contrary, as they alleged, to the terms of their agreement. In defence of his course, he said, "I am sure that such occurrences can scarce be too frequent, as it comforts the patient to think that he is with his family, and that they are affectionate to him; and upon inquiry of patients who have been cured of the same indisposition, they have always mentioned those occurrences having given them the greatest comfort, and, as they thought, helped very much towards their recovery. . . . The irritation occasioned by a patient's seeing his friends or relations is entirely overbalanced by the softening him into tears, which ever leads to amendment." In this statement of Willis, we may recognise the views of one of our early associates, the first President of this Society, between whom and Willis this was not the only point of resemblance.

Another incident in Willis's management, which had greatly scandalized his colleagues, was deemed worthy of the notice of the committee. It was the allowing his Majesty to read the tragedy of Lear. It seems he refused the King's request to have it, though too crazy, he thought, to be affected by it, one way or the other; but allowed him to have a volume of plays, which happened, without his knowledge, to contain Lear.*

In the practical knowledge of insanity, and the management of the insane, Willis was unquestionably in advance of his associates; but following the bent of his dictatorial habits, he often spoke without measuring his words, and often overstepped the limits of professional etiquette. Hence he suffered under the severe handling of the committee, to whom he presented a good by the EYE! I should have looked at him *thus*, sir—thus!" whereupon Burke instantaneously averted his head and made no reply. This must have occurred, if at all, in the committee-room, but no mention of it is made in the printed report. It may have been expunged, however, by the committee. What the common practice is, I am unable to say; but that such a thing is sometimes done, we have the authority of Sir Samuel Romily for believing. He states that some of the testimony of the physicians, in 1810, to the effect that the cause of the King's illness in 1801 was the resignation of Pitt, and the cause of the attack in 1804 was the publication of the correspondence between the Prince of Wales and the Duke of York, was suppressed. ["Memoirs," &c., ii, 165.] The authority for this anecdote is Reynolds, the playwright, who says he had it from Willis himself. ["Life," &c., ii. 15.] Among the gossip of the day was a similar story respecting the effect of Willis's tone on Sheridan when about to examine him. "'Pray, sir, before you begin,' said Willis, 'be so good as to snuff the candles, that we may see clear, for I always like to see the face of the man I am speaking to?' Sheridan was so confounded at this speech of the basilisk Doctor, that he could not get on in his examination, and for once in his life he was posed."—*Swinburne's Courts of Europe*, ii, 75.

* Willis's statement that he had never read this play, is not calculated to raise our estimate of his general culture.

many vulnerable points of attack. It is obvious, in fact, that Willis was a bit of a charlatan, and not always above the arts of that character. Sheridan remarked, in one of his speeches, that Willis professed to have the gift of seeing the heart by looking at the countenance; and added, looking at Pitt, that the declaration seemed to alarm the right hon. gentleman.*

But, with all these imperfections, it cannot be denied that Willis evinced much practical sagacity in his views of the nature and management of mental disease, and a sturdy independence and self-reliance which, while they are always elements in a great character, were in him, under the circumstances, little less than wonderful. Let those who are emulous of his success strive to imitate him in these qualities, rather than in his dogmatism and disregard of professional observances.

The report of the committee was a fruitful topic in the subsequent debates in parliament, furnishing fresh materials for declamation and intrigue. On no other occasion, probably, were the prominent qualities of the celebrated men who figured at that period more strikingly exhibited. Night after night, for weeks together, witnessed the unrivalled self-possession of Pitt—the clear, close, vehement argumentation of Fox—the irresistible wit of Sheridan—the multifarious knowledge and riotous fancy of Burke. But the prize, which seemed to be almost within the grasp of the Whigs, rapidly receded from their view. Towards the last of January the King had unquestionably improved, and on the 25th of February Warren signed a report declaring him "free from complaint."

The question of recovery was also embarrassing, for although it might be obvious enough to the family and friends, yet it was not so easy to establish it satisfactorily to the country. An apparent recovery is not always a real one. Often, after a person seems to have regained his natural feelings and views, and has recognised his mental disorder, and is preparing, perhaps, to resume his customary pursuits, he again passes under the cloud, and, to all appearance, is as far from sanity as ever. Burke was as ready for this as for any other occasion, and his remarks upon it exhibited his wonderful faculty of acquiring and appropriating every description of knowledge. "The disorder," said he, "with which his Majesty was afflicted, was like a vast sea which rolled in, and at low tide rolled back and left a bold and barren shore. He had taken pains," he continued, "to make himself master of the subject, he had turned over every book upon it, and had visited the dreadful mansions where

* There is nothing of this kind in the report of the committee, but it may have been suppressed. Sheridan would hardly have invented the fact, and then called on Pitt to witness its truth.

those unfortunate beings were confined. . . . . An author of great authority having mentioned the uncertainty of the symptoms of sanity, had declared, that after having been kept a month, (and the rule was at all the houses he had visited, though anxious to discharge the patients speedily, as they all were, to keep them a month after their recovery before they turned them out of the house,) they would sometimes dread the day of their departure, and relapse on the very last day. . . . He drew a picture of the King's supposed return, which he described as most happy, if really cured; but as horrible in the extreme, in its consequences, if a sudden relapse took place."

The only effect of the King's alleged convalescence was to suspend all parliamentary proceedings relative to a regency, while, quietly and without opposition, he resumed, one after another, his regal functions.*

* It may be a matter of surprise, at first sight, that, considering the disagreement between Willis and his colleagues respecting the signs of convalescence, some other physician of eminence in this branch of the art was not called in. "Why," said Burke, "is not the keeper of one mad-house confronted with the keeper of another?" referring to Monro, who then visited Bethlehem. It is probable, however, that the Government suspected—very justly too—that the measure, while it would certainly introduce a new element of discord into the medical councils, might not so surely strengthen their position.

Willis was rewarded by parliament with a pension of 1500*l.* for twenty-one years. He was shortly after employed to treat the Queen of Portugal, but she proved to be incurable. For this service he received 20,000*l.* These fees are without a parallel in the records of the medical profession. Dr. John Willis received for his services 650*l.* per year during his life.

It is somewhat calculated to abate our confidence in history, to find that so recent and public a fact as the result of Willis's treatment of this case should be related in such a contradictory manner. By many, if not the most of those who refer to it, including even such respectable authorities as the "Biographie Universelle" and "Penny Cyclopædia," it is represented to have been a complete cure. But the truth is—and obvious enough, too, it might seem—the poor Queen, who had been for some time hovering on the verge of insanity, became unequivocally deranged in 1792, and so continued without any improvement. In the early stage of her disease, she conceived the idea that she was doomed to eternal perdition. Her son, the Prince of Brazil, assumed the regency in 1792. In 1807, when the kingdom was invaded by the French, she followed the fortunes of her house across the ocean, though much against her will, and finally died in 1816, aged eighty-one.

In Frederick Reynolds's "Life and Times" I find a notice of Willis's establishment, which seems to be worth copying:—"Gretford and its vicinity at that time exhibited one of the most peculiar and singular sights I ever witnessed. As the unprepared traveller approached the town, he was astonished to find almost all the surrounding plowmen, gardeners, threshers, thatchers, and other labourers, attired in black coats, white waistcoats, black silk breeches and stockings, and the head of each '*bien poudré, frisé et arrangé.*' These were the Doctor's patients; and dress, neatness of person, and exercise being the principal features of his admirable system, health and cheerfulness conjoined to aid the recovery of every person attached to that most valuable asylum. The Doctor kept an excellent table, and the day I dined with him I found a numerous company. Amongst others of his patients, in a state of convalescence, present on this occasion, were a Mrs. B., a lady of large fortune, who had lately recovered under the Doctor's care, but declined returning into the world, from the dread of a relapse; and a young clergyman, who occasionally read service and preached for the Doctor. Nothing occurred out of the common way till soon after the cloth was removed, when I saw the Doctor frown

His Majesty's third attack began about the 22nd of February, 1801, and though supposed by the public to have recovered within three or four weeks, it is certain that he was not fully restored until the last of June. He was attended by Drs. Gisborne, Reynolds, Pepys, Robert Darling Willis, John Willis, and Thomas Willis.* The early stage of the disease was much like that of 1788, except in being of shorter duration. After the first week or two, he could, for the most part, control his morbid manifestations to such a degree, that, to them who saw him only occasionally, he seemed to be less under the influence of disease than he really was. Indeed, as early as the 7th of March, it was commonly reported, and commonly believed, that he had completely recovered, though on the 4th Reynolds had stated that "much time would be necessary to complete the cure."† The bulletins ceased on the 12th of March, when Reynolds ceased his attendance; but on the 14th or 15th of the same month he had a "severe paroxysm," as it was called, which, however, must have soon abated, as he transacted business on the 17th. He continued under medical care until the end of June, appearing very well whenever circumstances required the exercise of self-control, but constantly exciting the apprehensions of his family and physicians by some manifestation of mental disturbance. John Willis, writing to Lord Eldon, May 16th, intimates that "artificial prudence" is still absolutely necessary, and informs him that his conversations with the King have not been of much service. "He seems," he continues, "rather to select and turn any part to his purpose than to his good."‡ Five days after, Addington writes to Lord Eldon, that "during a quiet conversation of an hour and a half, there was not a sentiment, a word, a look, or a gesture, that I could have wished different from what it was; and yet my apprehensions, I must own to you, predominate. The wheel is likely to turn with increasing velocity, (as I cannot help fearing,) and if so, it will very soon become unmanageable."§ Four days after, one of the Willises writes, that the King "is in a perfectly composed and quiet state. He told me, with great seeming satisfaction, that he had had a most charming night—'but one sleep from eleven to half-past four;' when, alas! he had but three hours' sleep in the night, which, upon the whole, was passed in restlessness, in getting out of bed, opening the shutters, in praying at times at a patient, who immediately hastened from the room, taking with him my *tail*, which he had slyly cut off."

* Robert and John Willis were sons of Francis, and probably Thomas also, but of this I am not quite certain.

† Diaries of Lord Malmesbury, iv. 28.

‡ Twiss—Public and Private Life of Lord Chancellor Eldon, i. 204.

§ Ibid. i. 205.

violently. . . . He frequently called, 'I am now perfectly well, and my queen, my queen has saved me.' . . . . The King has sworn he will never forgive her if she relates anything that passes in the night."* June 9th, one of the royal family writes to Thomas Willis, "He has been very quiet, very heavy, and very sleepy. . . . . God grant that his eyes may soon open, and that he may see his real and true friends in their true colours." Three days after, she again writes, that "the sleepiness continues to a great degree. I am told the night has been tolerable, but he has got up in his usual way, which is very vexatious."† Four days after, one of the Willises writes: "His Majesty rode out this morning at ten o'clock, and did not return till four. He paid a visit in the course of the day to Mr. Dundas. His attendants thought him much hurried, and so did his pages. He has a great thirst upon him, and his family are in great fear. His Majesty still talks much of his prudence, but he shows none. His body, mind, and tongue are all upon the stretch every minute; and the manner in which he is now expending money in various ways, which is so unlike him when well, all evince that he is not so right as he should be."‡

A considerable change seems to have occurred within a few days of the date of this letter, since his physicians were discharged, and we hear no more of his disorder. He was strongly averse to having the Willises any longer about him, though, as he says, "he respected the character and conduct of Robert Willis." "No one," he says, "who has had a nervous fever can bear to continue the physicians employed on the occasion.§

During the first three weeks of the attack there was actually a suspension of the royal functions, and with it a suspension of some political arrangements of the highest importance. Pitt had resigned, but there was no one to receive his resignation, or sign the commission of his successor; so that it would have been difficult to answer the question, who is now prime minister? Pitt and his friends continued to perform the necessary routine duties of their offices, and Mr. Addington held constant communication with the palace.|| This change of ministry, which was exceedingly distasteful to the King, was regarded by some as the exciting cause of this attack; but it is probable that the differences between the Prince of Wales and his wife had also much

* Twiss, i. 205. † Ibid. i. 206. ‡ Ibid. i. 208.

§ The only thing respecting the medical treatment in this attack which has rewarded my inquiries is, that the prime minister, Mr. Addington, one day, recommended a hop pillow for procuring sleep, which proved perfectly successful. "In this attack sleep always calmed and quieted the King, while in that of 1788 he would awake from a long sleep more turbulent than ever."—*Malmesbury's Diaries*, iv. 46.

|| Life, &c., of Lord Sidmouth, by Pellew, i. 309.

to do with it. It was ushered in by a violent cold, which he contracted by remaining long in church on the 13th—a chilly, snowy day.

Again, on the 12th of February, 1804, the King manifested unequivocal signs of mental disease, occasioned, it was thought, by the publication of certain correspondence between the Prince of Wales and the Duke of York, and immediately preceded by a cold and a consequent fit of the gout. This attack continued longer than the last, but, like that, was much less severe than the attack of '88. He was attended by Sir Lucas Pepys, Dr. Reynolds, Dr. Heberden, and Dr. Simmons, physician of St. Luke's,* and was in the particular charge of the latter, who resided in the palace. The few scanty notices I have been able to find convey but little information respecting the character or progress of this attack. About the 25th of February it was generally understood that the King was improving; but in the bulletin of the 26th, it was stated that his speedy recovery could not be expected.† We learn that, on the 9th of March, Lord Eldon walked with him around the garden, when he observed, as he says, "at first, a momentary hurry and incoherence in his Majesty's talk, but this did not endure two minutes; during the rest of the walk there was not the slightest aberration in his Majesty's conversation, and he gave me the history of every administration in his reign."‡ On the 23rd of April, he presided at a council. On the 2nd of May, Addington walked with him in the garden, and thought him perfectly well.§ Five days after, Pitt conversed with him three hours, and was "amazed at his cool and collected manner."|| May 25th, the Duke of York writes that the King seems to dwell much upon the illegality of his confinement; and the next day, Pitt, in a note to Eldon, expresses some alarm in reference to a conversation in one of the audiences two days before. "The topics treated of were such as did not at all arise out of any view (right or wrong) of the *actual state* of things, but referred to plans of foreign politics,

* Why none of the Willises were employed on this occasion does not appear. It was probably, however, for the same reason that was alleged for their not being employed in the next attack—viz., the Queen's apprehension that their presence would excite unpleasant associations in the King's mind. In fact, the King conceived a strong dislike for the Willises; but it seems to have been a common impression at court, [Malmesbury, iv. 316,] that they managed him much better than Simmons.

† Bulletins must necessarily be brief, and very general in their terms, and therefore not calculated to convey very accurate information; but those which were issued by the physicians during this illness often indicate much confusion of ideas, and an uncertain, vacillating prognosis, which did not escape the notice nor the censure of parliament. For instance, the very next day after the bulletin above mentioned, the bulletin said, "He is still better than he was yesterday, and gradually approaching recovery."

‡ Twiss, i. 223. § Life of Sidmouth, i. 313. || Malmesbury, iv. 306.

that could only be creatures of an imagination heated and disordered."*

His conduct at this period, as described by one of his court, indicates a phasis of insanity which, though common enough, is apt to be greatly misunderstood by people not professionally acquainted with the subject. "Mrs. Harcourt confirms all that Lady Uxbridge had told me—that the King was apparently quite well when speaking to his ministers, or to those who kept him in a little awe; but that towards his family and dependents his language was incoherent and harsh, quite unlike his usual character. She said Simmons did not possess, in any degree, the talents required to lead the mind from wandering to steadiness;—that, in the King's two former illnesses, this had been most ably managed by the Willises, who had this faculty in a wonderful degree, and were men of the world, who saw ministers, and knew what the King ought to do;—that the not suffering them to be called in was an unpardonable proof of folly (not to say worse) in Addington; and now it was impossible, since the King's aversion for them was rooted;—that Pitt judged ill in leaving the sole disposal of the household to the King;—that this sort of power, in his present weak, and, of course, suspicious state of mind, had been exercised by him most improperly: he had dismissed and turned away, and made capricious changes everywhere, from the Lord Chamberlain to the grooms and footmen; he had turned away the Queen's favourite coachman, made footmen grooms, and *vice versâ*, and what was still worse, because more notorious, had removed lords of the bedchamber without a shadow of reason;—that all this afflicted the royal family beyond measure; the Queen was ill and *cross*—the Princesses low, depressed, and quite sinking under it;—and that, unless means could be found to place some very strong-minded and temperate person about the King, he would either commit some extravagance, or would, by violent exercise and carelessness, injure his health, and bring on a deadly illness. . . . . She said that Smart, when alive, had *some* authority over him;—that John Willis also had acquired it, but in a different way: the first obtained it from regard and high opinion, the other from fear:—that, as was always the case, cunning and art kept pace, in the King's character, with his suspicion and misgivings, and that he was become so very acute that nothing escaped him."†

The general impression at the time was, that, in both these attacks, the King was deprived of his reason for a short period only; and parliament was readily satisfied by the declarations of ministers, that there was no necessary suspension of the royal functions. Before the question of a regency could be fairly

* Twiss, i. 241. † Malmesbury, iv. 326.

started, the bulletins ceased, and he was supposed to have recovered. Of course, there was no examination of the physicians, and the public had no means of learning the subsequent progress of the disorder, because they alone to whom the facts were known were most interested in keeping them to themselves. It was not until the examination of the physicians, relative to the next attack (1810), some of whom had also attended him in 1801 and 1804, that the true state of the case was revealed.* It then came out for the first time, that both these attacks were of much longer duration and greater severity than the public had been led to suppose,—that, about the middle of March, 1801, and after the bulletins ceased, a relapse took place,—that, in 1804, Dr. Simmons continued in the palace as late as June,—and that either Heberden or Sir Francis Millman attended the King up to October.† And yet it had become a matter of history, that during those very periods when his Majesty was in charge of medical men on account of mental disorder, he was exercising the highest functions of sovereignty. On the 17th of March, 1801—which, as we have just seen, was only two or three days subsequent to the date of a "severe paroxysm"—measures of vital interest and importance to the country received his assent and concurrence. On the 14th of April, Pitt's resignation was accepted, and the new ministers received their commission. On the 9th of March, 1804, a commission, under the King's sign-manual, was passed, by virtue of which fifteen bills received the royal assent, and, on the 23rd, his assent was given to many other bills.

It is not surprising that the discovery of his real mental condition, half a dozen years afterwards, excited both astonishment and indignation. In parliament, the conduct of Lord Eldon, who, in consequence of his office as Lord Chancellor, and of his intimate personal relations to the King, was held responsible for these transactions, was condemned in the strongest terms. Earl Grey charged him with having done what was equivalent to treason. "What," said he, "would be the character, what the appropriate punishment of his offence, who, knowing his Sovereign to be actually at the time incompetent,—who, in the full conviction of his notorious and avowed incapacity, and whilst he was under medical care and personal restraint, should

* It must be borne in mind that the memoranda showing the progress of the disease, which we have given, were mostly published only a few years ago, so that, in fact, the whole state of the case was not generally known even after the examination of the physicians in 1811.

† Indeed, as late as December, the King had not entirely regained the confidence of his family. Lord Malmesbury says, (iv. 344,) on the authority of one of the court, "The Queen will never receive the King without one of the Princesses being present,—never says, in reply a word,—piques herself on this discreet silence,—and, when in London, locks the door of her *white room* (her boudoir) against him."

come here, and in the name and under the pretext of his Majesty's commands, put the royal seal to acts which could not be legal without his Majesty's full and complete acquiescence?" . . . . "I will ask the noble lord," he continued, in another part of his speech, "what he would have done, had a case of a similar nature come before him in Chancery? I will suppose such a case; and that, in the interval, when it appeared from the testimony of physicians that the unfortunate individual was incapable of exercising his mental faculties, a person had prevailed on an attorney to make a will for him; would the noble lord have given his sanction to such a proceeding? Would he have taken the opinion of the interested individuals, in preference to that of the physician? Let the noble lord apply this case to himself. I say that his Majesty's name has been abused. The noble lord has said, on his own authority, that his Majesty was not then incapacitated from acting; but will your lordships allow yourselves to believe that his Majesty's health was then such as to admit him to act in his royal incapacity, upon an authority which contradicts that of his physicians?"

In his defence, Lord Eldon declared, that on the 27th of February, and again on the 9th of March, 1804, the King's physicians had pronounced him competent to perform a certain act; or, as the matter was described more particularly in his Memoirs, he inquired of the physicians if, in their opinion, the King was competent to sign an instrument, provided he, Lord Eldon, had satisfied himself that the King understood its effect. To this query Sir Lucas Pepys and Dr. Simmons replied affirmatively, the other physicians being supposed to concur. Chiefly, however, he grounded his defence on the right to judge for himself respecting the King's mental condition, irrespective of medical opinions. "I have been significantly asked," said he, "if I would supersede a commission of lunacy against the opinion of physicians. I have often done so. The opinions of physicians, though entitled to great attention, were not to bind him absolutely. . . . . It was most important to the Sovereign that the Chancellor should not depend wholly on the evidence of the physicians, if he himself thought the King perfectly competent to discharge the functions of the royal authority."* In a letter to Percival, he declares that if the King had been found to understand the nature of the act he was asked to perform, he should have been bound by his sense of right and duty to have sanctioned such act, though he might have believed, with his physicians, that some delusions might occur an hour afterwards.†

Eldon declared, in the debate, that, on the 9th of March,

* Stockdale's Parliamentary Register, 1811, i. † Twiss, i. 356.

1804, the King understood the duty he had to perform better than he did himself, and among his papers was found what he regarded as a conclusive proof of his opinion. "On applying to the King," he says, "to obtain his sign-manual to several bills, he, Eldon, began to read an abstract of the bills with more of detail than usual, when the King said, 'My lord, you are cautious.' He, Eldon, begged it might be so, under existing circumstances. 'Oh!' said the King, 'you are certainly right in that; but you should be correct as well as cautious.' Eldon replied, he was not conscious that he was incorrect. 'No,' said he, 'you are not; for if you will look into the commission you have brought me to sign, you will see that I there state that I have fully considered the bills proposed to receive my sign-manual. To be correct, therefore, I should have the *bills* to peruse and consider.' I stated to him that he had never had the bills whilst I had been chancellor, and that I did not know that he had *ever* had the bills. He said, during a part of his reign, he had always had them until Lord Thurlow had ceased to bring them; and the expression his Majesty used was, Lord Thurlow said it was nonsense his giving himself the trouble to read them."*

Lord Eldon, as well as the physicians, made the common mistake of confounding the power to understand the exact terms of a transaction, with that of perceiving all its relations and consequences. Such a mistake, natural enough as it might have been to him, could hardly have been expected from the physicians, especially under circumstances so peculiar and important. It would be considered a bold assertion, that a person regarded by his family and physicians as insane was perfectly competent to make a contract or execute a will; but to declare that the King, who, by their own admission, was more or less insane, was, nevertheless, competent to exercise the most important functions of his office, was, to say the least of it, to assume a tremendous responsibility. But they knew very well the wishes of the Court on the subject; and it could hardly have been expected of Court physicians that they would be over-scrupulous on such an occasion, especially as they were aware, no doubt, that the measures in question were proper enough in themselves, and the royal assent was merely a matter of form. This, unquestionably, was the real ground on which Eldon acted, though it did not furnish the kind of defence exactly which he was disposed to set up. The nation was at war; a change of ministry was in progress, both in 1801 and in 1804; a project of a regency would have distracted the national councils and impaired the national vigour; and the disease, scarcely severe at any time, seemed likely to be of very short duration. A man

* Ibid. i. 226.

much less devoted to political ends than Eldon might, under such circumstances, have considered it perfectly justifiable to avoid the real evils of a regency question by committing one more theoretical than practical, and followed by salutary consequences. In fact, the same thing was done by Lord Loughborough, who went to his Majesty on the 24th of February, 1801—Addington having declined the service—and obtained his signature to a commission for giving the royal assent to the Brown Bread Bill.*

There was another charge against Lord Eldon, which cannot be so easily parried. It was insinuated by Earl Grey, in the debate already alluded to, that he used the facilities of his position to prevent a junction between Fox and Pitt in 1804; and it appears, from his own papers, that he used similar means to accomplish the removal of Addington, his own colleague, and bring in Pitt. These might have been precisely the arrangements which the King would have favoured, had his mind been perfectly sound; but no man could have promoted them as Eldon did, without forfeiting every claim to upright and honourable conduct.†

About the 25th of October, 1810, the King was again, and for the last time, smitten by mental disease, consequent, it was generally supposed, upon the fatal illness of a favourite daughter. It began, like the former attacks, with unusual hurry and restlessness of manner, which, within a few days, passed into a paroxysm of high excitement, accompanied by much fever. During the first few months the disorder was characterized by paroxysms of this kind—in one of which he is said to have been "unconscious of surrounding objects"—alternating with intervals, when the King was free from fever, calm, composed, and quite rational in his conversation. He was attended by Reynolds, Heberden, Baillie, Halford, and Robert Willis, the latter residing in the palace and having the immediate custody of the King, as his father had in 1788. The physicians were examined by a committee of the Commons on the 14th of December, and by a committee of the Lords about the same time. The questions propounded were precisely the same as those of 1788, and the replies were of a very similar character. They all concurred in the opinion that the disease would ultimately yield, but no one

* Life of Lord Sidmouth, i. 302.

† True, Eldon pronounced the charge, that he had taken advantage of the King's weakness to prejudice him against Mr. Fox, to be a direct falsehood. His biographer candidly remarks, that this "denial must not be extended beyond the charge it was meant to meet, of having taken advantage of the King's weak state to excite a prejudice against Fox in the royal mind"—meaning probably. that, as he did not believe the King to be incompetent, he might safely deny that he took any advantage of his weakness.—*Twiss*, i. 356.

undertook to set limits to its duration. The same reasons, too, were also given for this favourable prognosis—the patient's previous good habits and firm health, the suddenness of the attack, and the general curability of the disease. To the question, whether his Majesty's age, then seventy-two years, was not an unfavourable circumstance, the unanimous answer was, that, as a general rule, extreme age was an unfavourable circumstance, in mental as well as other diseases; but, in the present case, it would probably have little influence upon the result, because the King had borne his age remarkably well, and the attack had originated in circumstances independent of any bodily indisposition. To the question, whether the King's very defective sight—for he had become almost, and soon after entirely, blind—might not operate unfavourably, the reply was, substantially, that, in the early stages of the disorder, it would be more likely to have a beneficial effect than otherwise, by keeping from him many sources of irritation; while, in the later stages, it might, by diminishing his means and opportunities of occupation, retard his recovery. To the question, whether the fact of his having had so many previous attacks was not an unfavourable circumstance, Reynolds and Baillie replied—to them only was the question put—that his having recovered from so many previous attacks, furnished strong grounds for expecting recovery again. Baillie, however, qualified his opinion by the suggestion that the susceptibility to disease might be increased by its frequent recurrence, and thus prove an obstacle to recovery.

In regard to the form of disease, Willis said it was more allied to delirium than insanity—meaning that it was characterized by mental excitement rather than by fixed, definite delusions: "It has never borne the character of insanity," he said: "it never gets beyond derangement." This description, he added, was strictly applicable to the attack of 1801. Heberden said: "It is not merely the delirium of fever, nor is it any common case of insanity; it is derangement attended with more or less fever, and liable to accessions and remissions." The form of disease which they had in view is common enough; and though the progress of science may have contributed nothing to our knowledge of nature or of its treatment, it certainly has improved our nomenclature.*

The Report conveys no information respecting the medical or moral treatment, and we are left in doubt whether mechanical restraint was used. In fact, the examination was chiefly directed, not so much to the present condition of the King as to the attacks of 1801 and 1804, several of the physicians having

* The Report may be found in Stockdale's Parliamentary Register, 1810, and Hansard's Parliamentary Debates, 1st ser. xix.

attended him at one or both those periods, and to some interviews between the King and his ministers. It showed the usual amount of intrigue and cabal on the part of the King's friends, with subserviency to the predominant party and disregard of each other on the part of the physicians. As in the illness of 1788, the policy of the Tories was to stave off the regency by representing the attack as speedily curable, while the Whigs were equally strenuous in precipitating this measure. But the result appeared so doubtful, and the exigencies of the country were so pressing, that it could not long be evaded; and, accordingly, the Prince of Wales was made Regent in February, 1811 —an event which enabled the Whig party, as is well known to all who are acquainted with the history of that period, to verify the scriptural declarations respecting the faithlessness of princes.*

The progress of the disease may be gathered from casual notices in the memoirs, correspondence, diaries, &c., of the time, but not so exactly as it might be on some interesting points. On the 26th of January, Eldon spent an hour with him. "He is not well," says the Chancellor, "and I fear he requires time. In the midst of this state it is impossible to conceive how right, how pious, how religious, how everything that he should be, he is, with the distressing aberrations I allude to."† In his clearer intervals he became somewhat impatient of restraint, and was rather importunate to be restored to his regal state. The physicians, in their report to the Chancellor, which must have been about the first of February, say that "he appears to be going on in the most favourable manner. It is right to mention, and we do not think it an unfavourable circumstance, that he has occasionally adverted to the subject of his former delusion, but in so slight a manner as to increase our confidence in its gradual subsidence from his Majesty's mind."‡ The Queen, in a note to Lord Eldon, soliciting the attendance of one of the council at Windsor at least once a week, says: "The King is constantly asking if not one of the council is coming to do so, [to receive the report of the physicians,] and seems to feel that putting it off procrastinates his recovery, as his Majesty (*she is sorry to say*) thinks himself too near that period."§ Spring brought no improvement of the King's disorder. In a note of Lord Ellenborough, April 3rd, he speaks of the King's "delusions and irregularities, and extravagances of plans and projects, of which we hear daily."|| May 25th, the Duke of York had an inter-

* Romilly (Memoirs, ii. 177) says that the Prince was determined to make no change in the cabinet, in consequence of the strong representations of one of the King's physicians of the probability of his recovery.

† Twiss, i. 359. ‡ Ibid. i. 359. § Ibid. i. 359. || Ibid. i. 363.

view with him, in which his mental condition was pretty fairly exhibited. "He appeared," he says, "at first, very much affected at seeing me, and expressed himself in the kindest and most affectionate manner upon my re-appointment to the chief command of the army; but soon flew off from that subject, and then ran on, in perfect good humour, but with the greatest rapidity, and with little or no connexion, upon the most trifling topics, at times hinting at some of the subjects of his delusion, in spite of all our endeavours to change the conversation."* Robert Willis expressed to the Duke his alarm at the King's "frivolity, or rather imbecility, of mind."

Until July, the cloud which enveloped the mind of the King occasionally lifted up, and thus were strengthened the hopes of his complete restoration. It was one of the curious traits in his case, that, at those times, he became conscious of his infirmity, though he sometimes manifested this consciousness in rather an uncommon manner. An instance is related by Francis Horner, in a letter to his father, in the spring of 1811. "There was a very affecting proof of the King's melancholy state given last week at the concert of ancient music; it was the Duke of Cambridge's night, who announced to the directors that the King himself had made the selection. This consisted of all the finest passages to be found in Handel descriptive of madness and blindness, particularly those in the opera of Samson; there was one also upon madness from love, and the lamentation of Jephtha upon the loss of his daughter; and it closed with 'God save the King,' to make sure the application of all that went before."†

Dr. Simmons and Dr. John Willis, who had attended the King in former attacks, had not been employed in this, the Queen fearing that it might awaken disagreeable emotions. A year having passed without any improvement, these two physicians were joined to the medical corps on the 9th of October, together with Dr. Monro, then visiting physician at Bethlehem. They were all examined touching the King's condition, both by a committee of the Lords and a committee of the Commons, towards the middle of January, 1812.

From this examination we gather that, during the months of April, May, and June, the King was apparently improving, "very little disorder being exhibited," says Heberden. It was characterized by exaltation, extravagance, and frivolity—false reasoning upon real facts. About the middle of July the disorder assumed a new character, gross delusions being exhibited in connexion with the last-mentioned traits. His sight and hearing were quite gone, but the theor senses were as acute as

* Twiss, i. 363. † Memoirs and Correspondence, ii. 70.

ever. He retained a consciousness of his regal state, and during the latter part of the year, when there seemed to be a little improvement, he bore his part in conversation very correctly, for a few minutes, and related anecdotes of the past. The physicians were all as confident in the opinion that his recovery, though not hopeless, was highly improbable, as they were, the year before, in the opinion that he would recover. This change in their prognosis they attributed chiefly to the change in the phasis of the disorder, which occurred in July.*

This report leaves us entirely in the dark respecting the nature of the delusions which possessed the King's mind, but the following passage from Lord Eldon's papers indicates one of them. "It was agreed that, if any strong feature of the King's malady appeared during the presence of the council, Sir Henry Halford should, on receiving a signal from me, endeavour to recal him from his aberrations; and, accordingly, when his Majesty appeared to be addressing himself to two of the persons whom he most favoured in his early life, long dead, Sir Henry observed, 'Your Majesty has, I believe, forgotten that —— and —— both died many years ago.' 'True,' was the reply, 'died to you and to the world in general, but not to me. You, Sir Henry, are forgetting that I have the power of holding intercourse with those whom you call dead. Yes, Sir Henry Halford,' continued he, assuming a lighter manner, 'it is in vain, so far as I am concerned, that you kill your patients. Yes, Dr. Baillie —— but, Baillie, Baillie,' pursued he, with resumed gravity, 'I don't know. He is an anatomist; he dissects his patients; and then it would not be a resuscitation merely, but a recreation, and that, I think, is beyond my power.'"†

The following memoranda of his condition from 1812 till his death are given by an anonymous writer, but are well authenticated, I believe, and comprise all that I have been able to find respecting this period. "At intervals he still took a lively interest in politics. His perception was good, though mixed up with a number of erroneous ideas; his memory was tenacious, but his judgment unsettled; and the loss of royal authority seemed constantly to prey upon his mind. His malady seemed rather to increase than abate up to the year 1814, when, at the time the Allied Sovereigns arrived in England, he evinced indications of returning reason, and was made acquainted with the astonishing events which had recently occurred. The Queen, one day, found the afflicted monarch engaged in singing a hymn, and accompanying himself on the harpsichord. After he had concluded the hymn, he knelt down, prayed for his

* Hansard, xxi. 73.

† Campbell's Lives of the Lord Chancellors, art. "Eldon," vii. 222.

family and the nation, and earnestly supplicated for the complete restoration of his mental powers. He then burst into tears, and his reason suddenly left him. But he afterwards had, occasionally, lucid moments. One morning, hearing a bell toll, he asked who was dead. 'Please your Majesty,' said an attendant, 'Mrs. S.' 'Mrs. S.!' rejoined the King, 'she was a linen-draper, at the corner of —— street, and brought up her family in the fear of God. She has gone to heaven: I hope I shall soon follow her.' He now became deaf, imbibed the idea that he was dead, and said, 'I must have a suit of black in memory of George III., for whom I know there is a general mourning.' In 1817 he appeared to have a faint glimmering of reason again; his sense of hearing returned more acute than ever, and he could distinguish persons by their footsteps. He likewise recollected that he had made a memorandum many years before, and it was found exactly where he indicated. After 1818 he occupied a long suite of rooms, in which were placed several pianos and harpsichords; at these, he would frequently stop during his walk, play a few notes from Handel, and then stroll on. He seemed cheerful, and would sometimes talk aloud, as if addressing some nobleman; but his discourse bore reference only to past events, for he had no knowledge of recent circumstances, either political or domestic. Towards the end of 1819 his appetite began to fail. In January, 1820, it was found impossible to keep him warm; his remaining teeth dropped out, and he was almost reduced to a skeleton. On the 27th he was confined wholly to his bed, and on the 29th of January, 1820, he died, aged 82 years."*

* "Georgian Era," i. No authority is given for the statements in this work, and I am unable to verify them.

It is a curious coincidence, that this monarch, who suffered so much from mental diseases, should have been pursued, as if by a kind of fatality, by insane people. In 1786, an old woman (Margaret Nicholson) attempted to stab him, as he was alighting from his carriage; in 1790, a lieutenant of the army (John Frith) threw a stone at him through the window of the carriage in which he was riding; and, in 1800, a soldier (James Hadfield) shot at him with a pistol in the theatre. Miss Burney says that, during his illness in 1788, they were often annoyed by insane persons, who contrived to elude the restrictions of the palace and to roam over the grounds. The persons who committed the first two assaults were so obviously insane that, without any further action, the Privy Council sent them to Bethlehem Hospital. Hadfield was brought to trial, and, it being on an action of treason, his counsel was allowed to speak in his defence; for, until quite recently, this privilege was never permitted in criminal cases, except those of treason. It was on this occasion that Erskine made his greatest forensic effort; and it is a fact that may abate our pride of progress, that it has never been equalled in the clear apprehension it displays of the phenomena of insanity, in its plain and cogent views of responsibility, and its triumphant demolition of those principles which had been regarded, from the earliest times till that moment, as the settled law of England respecting insanity.

Like everything connected with State affairs, the incidents of King George's attacks have been enveloped in secrecy and mystification, and hence the difficulty of distinguishing between the true and the false. Some of them are obviously fabulous, and, together with others less improbable, had their origin, undoubtedly, in that sort of gossip which would naturally spring from such an interesting event as the insanity of the sovereign. Considering that the purposes of this narrative could be answered only by the strictest historical accuracy, I have been careful, in every instance, to indicate the source of my materials, and to make use of none that could not be well authenticated. The necessity of this kind of caution can scarcely be appreciated by those who have never learned, from their own inquiries into past events, how the false, the fabulous, the exaggerated and the true become blended together beyond the power of the most patient research to separate. To relate a striking incident, or a pointed anecdote, is an easy and agreeable duty; but to search out the authority on which they rest—in other words, to perform a great deal of fruitless labour—is a task often difficult and disagreeable.

